# Evaluation of CRISPR/Cas9 Constructs in Wheat Cell Suspension Cultures

**DOI:** 10.3390/ijms24032162

**Published:** 2023-01-21

**Authors:** Krzysztof Michalski, Paulina Ziąbska, Sławomir Sowa, Janusz Zimny, Anna M. Linkiewicz

**Affiliations:** Plant Breeding and Acclimatization Institute-National Research Institute, Radzików, 05-870 Błonie, Poland

**Keywords:** genome editing, ABA 8′-hydroxylase, *Agrobacterium tumefaciens*, polyploids

## Abstract

Despite intensive optimization efforts, developing an efficient sequence-specific CRISPR/Cas-mediated genome editing method remains a challenge, especially in polyploid cereal species such as wheat. Validating the efficacy of nuclease constructs prior to using them in planta is, thus, a major step of every editing experiment. Several construct evaluation strategies were proposed, with PEG-mediated plasmid transfection of seedling-derived protoplasts becoming the most popular. However, the usefulness of this approach is affected by associated construct copy number bias and chromatin relaxation, both influencing the outcome. Therefore, to achieve a reliable evaluation of CRISPR/Cas9 constructs, we proposed a system based on an *Agrobacterium*-mediated transformation of established wheat cell suspension cultures. This system was used for the evaluation of a CRISPR/Cas9 construct designed to target the *ABA 8′-hydroxylase 1* gene. The efficiency of editing was verified by cost-effective means of Sanger sequencing and bioinformatic analysis. We discuss advantages and potential future developments of this method in contrast to other in vitro approaches.

## 1. Introduction

Genome editing using a clustered, regularly interspaced short palindromic repeats CRISPR-associated (Cas9) endonuclease (CRISPR/Cas9) technique provides significant opportunities for the improvement of cereals. However, examples of the successful generation of novel genotypes of bread wheat (*Triticum aestivum* L.) with CRISPR/Cas9 are still very limited due to various factors, namely the efficiency of T-DNA integration, the in vitro regeneration capacity of a given genotype and the high number of single nucleotide polymorphisms (SNPs) interfering with sgRNA annealing [1]. Some of these limitations can be overcome by optimizing vector construction [2,3,4,5], improving nuclease construct delivery into plant cells [6,7] and enhancing plant regeneration via the implementation of morphogenic factors [8,9]. Nonetheless, a system for assessing the efficiency of the target locus modification could improve the selection of CRISPR/Cas9 genetic constructs to be used for genome editing. For this purpose, plant cell suspension cultures (PCSCs) can be applied as they show many analogies in terms of physiological and biochemical characteristics of the whole plant. Cereal PCSCs are usually established from embryo [10,11,12] or anther-derived calli [13,14,15]. Some reports also mention the initiation of PCSCs from calli obtained from the leaf base or inflorescence [16,17]. Upon the transfer of a friable and fast-growing callus to a liquid medium, cell suspensions are usually established within 2–3 months. Plant regeneration from PCSCs can be also obtained—Biesaga-Kościelniak et al. [18] described a method of direct suspension culture initiation from wheat immature embryos, with a regeneration capacity of 30%. Haploid suspension with regeneration potential proves especially useful in plant transformation and other genetic applications [19].

PCSCs have recently been introduced as a model system for evaluating the effectiveness of sequence specific nucleases (SSN) for genome modification. They allow for an analysis of a given construct in stable conditions of in vitro cultures, thus improving experimental reproducibility. As such, PCSCs, similar to callus cultures [20], can be utilized in two different ways: as a stable and uniform source of protoplasts for transfection experiments [21], or as an explant source for a direct *Agrobacterium tumefaciens*-mediated transformation [22,23,24], allowing not only SSN testing but, in some cases, also a regeneration of edited plants [25]. No similar experiments were previously conducted on wheat, despite its significant role in global food production [26,27].

Here, we report the utilization of long-term wheat cell suspension cultures as a model for the evaluation of gRNA/Cas9 constructs delivered by *A. tumefaciens*. We show that cell suspensions can be easily multiplied and successfully used for transformation with the selected gRNA/Cas9 constructs. Our system was used for the assessment of editing efficiency of gRNA/Cas9 constructs designed to target the *ABA 8′-hydroxylase 1* gene. The on- and off-target activity of Cas9 in stably transformed suspension culture-derived callus lines was evaluated. With further development, the proposed system might prove an interesting alternative to the routinely used construct validation by protoplast transfection.

## 2. Results

### 2.1. Wheat Cell Suspension Production and Transformation

**Figure 1:**
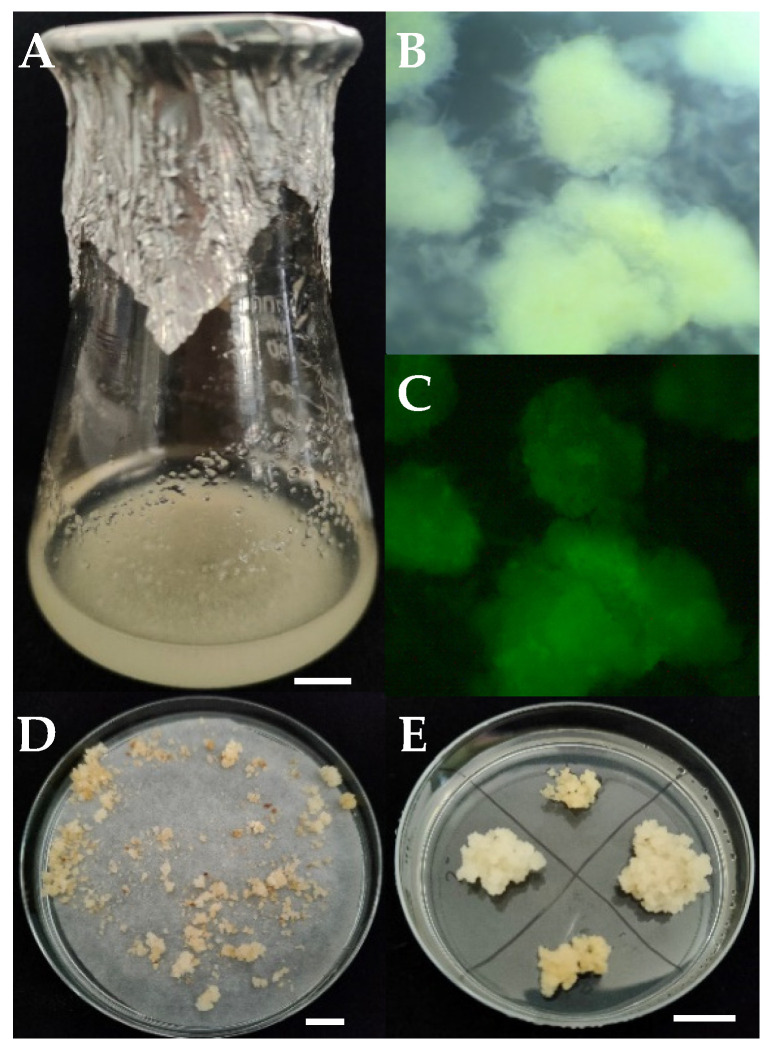
Subsequent stages of the wheat cell suspension transformation: (**A**)—an established suspension cell culture; (**B**)—cell aggregates; and (**C**)—a transient GFP expression 48 h after *A. tumefaciens* inoculation (36× magn.); (**D**)—primary growth of transgenic cell lines after 3 weeks on solid selection medium; (**E**)—proliferation of selected cell lines on solid selection medium. Bar 1 cm.

Wheat suspension cultures (Figure 1A) were established within three months. The six- to eight-week transformation and selection procedure (Figure 1B–E) provided 10 to 30 transgenic aggregates per 1 mL of the inoculated suspension. A successful transfer of T-DNA was confirmed 48 h after inoculation by microscope observation of green fluorescent protein (GFP) fluorescence. The selected aggregates (Figure 1D) proliferated successfully and were maintained on the solid medium with antibiotics (Figure 1E). Qualitative RT-PCR analysis confirmed Cas9 expression in the nine selected lines.

### 2.2. Evaluation of On- and Off-Target Editing Efficiency

**Figure 2:**
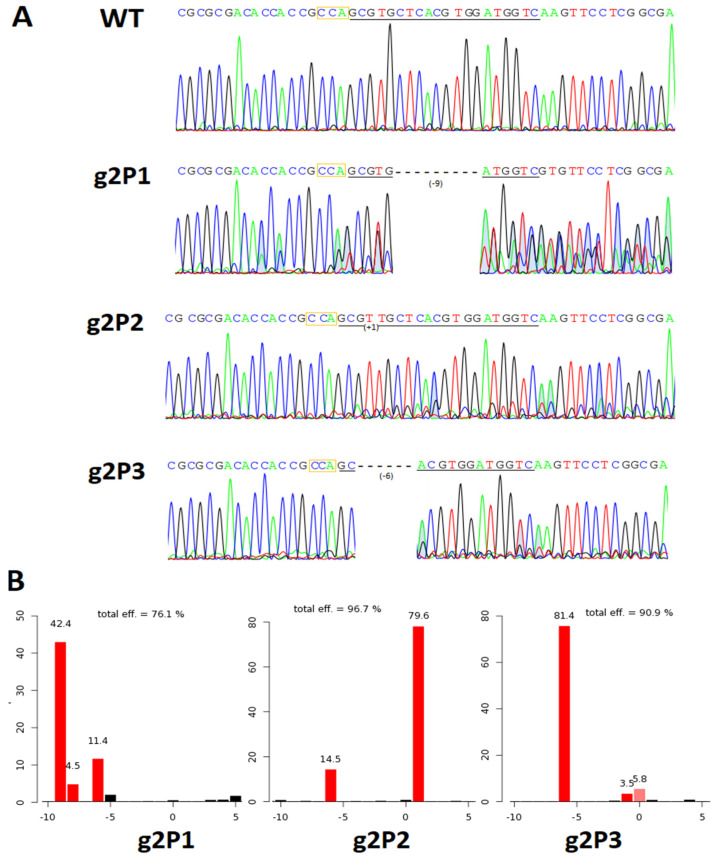
Example data set of chromatograms (**A**) and TIDE-generated indel spectra (**B**) of the most frequent edits in wheat sub-genome A target site in g2P1, g2P2 and g2P3 cell lines. (**A**): indel size in bp indicated by a number in brackets; protospacer underlined, PAM marked with the yellow frame; WT—wild type. (**B**): editing efficiency (%) indicated on *y* axis, indel size indicated on *x* axis. For more data please see the supplementary material Figure S1.

Three single aggregate-derived cell lines were tested and evaluated per each gRNA genetic construct (Table 1). We observed editing effects in all wheat cell lines tested. Small insertions or deletions of less than 10 bp were the most frequent edits (Figure 2A). Editing efficiency was defined as the summarized spectrum of indels and their frequency by the TIDE algorithm for all types of mutation. Efficiency varied between respective subgenomes, with the A genome being most frequently modified for all constructs tested, followed by the D genome and the B genome. For the gABA/1/364 guide, significantly higher editing efficiency was observed in cells transformed with the phosphinotricin construct (up to 75.5 ± 9.9%) in contrast with the hygromycin one (up to 33.1 ± 19.5%). The gABA/1/364 Phos was also the only combination that gave detectable edits in wheat subgenome B. Chromatograms and TIDE analysis revealed that each of our transgenic lines contained one or two predominant on-target mutations (*p* < 0.001) per homeolog locus (Figure 2B and Figure S1). 

We also tested two potential off-target sites [5] located on chromosomes 7B and 5A/5B/5D for gABA/1/364 and gABA/2/323, respectively. No detectable modification could be found at the tested off-target sites.

## 3. Discussion

Site-directed mutagenesis that proved to be efficient in model plants [28] remains problematic in wheat due to its polyploidy and low in vitro transformation–regeneration response [29,30]. Thus, the efficiency of gRNA/Cas9 constructs must be verified to estimate their potential efficacy in a plant editing experiment. Demonstrating the effectiveness of a system on a whole plant level may be a long and labor-intensive task; therefore, an alternative approach to assessing the efficiency of constructs for wheat editing should be elaborated [5,29,31].

PCSCs have long been recognized as a valuable tool to investigate various cellular functions at molecular, biochemical, and physiological levels in both model species as well as in cereals [32,33,34]. Individual cell culture systems have been used for genetic engineering studies in cereals since they are more amenable to DNA delivery and, in some cases, plant regeneration.

Here, we present the results of the gRNA/Cas9 construct evaluation through the stable transformation of wheat cell suspension cultures. We show that mutations in the *ABA 8′-hydroxylase 1* gene induced by gABA/1/364 Phos were present in all wheat subgenomes (A, B, and D). On the other hand, when hygromycin was used for selection, we did not detect mutations in the B subgenome. The construct harboring gABA/2/323 and phosphinotricin selection produced mutations solely on A and D subgenomes. The overall detected mutation rates ranged from 6.9% to 97.7%.

With the gRNA/Cas9 delivery via *A. tumefaciens* and its stable integration into the wheat genome, we observed differences in the nuclease efficiency between distinct subgenomes, despite the fact that both gRNAs fully match their on-target sites in the respective homeologs. No such differences in indel frequencies were detected when the same constructs were delivered into triticale (xTriticosecale Wittmack) protoplasts via polyethylene glycol (PEG)-mediated transfection, where editing efficiency in the presence of *TREX*2 reached, in all subgenomes, comparable levels of up to 53.5% and 44.2% for gABA/1/364 and gABA/2/323 gRNAs, respectively [5]. We also conclude that the hygromycin-based selection regime is less stringent and probably leads to a higher number of non-transgenic escaped cells.

Our observations raise questions about the precision of the gRNA/Cas9 delivery and evaluation methods, i.e., protoplast transfection mediated by PEG versus *Agrobacterium*-mediated transformation of cell suspensions. Discrepancies in editing outcomes observed between these approaches can be explained by a dosage effect, since PEG-mediated cell transfection introduces considerably more copies of foreign DNA than *Agrobacterium* does. This increased copy number then leads to particularly high levels of functional nucleases, facilitating short-term editing activity that is probably not achievable by a stable transformation. Furthermore, the chromatin accessibility in recipient cells might be another explanation for the condition. Xu et al. [35] showed that the protoplast isolation procedure leads to a genome-wide relaxation of chromatin in *Arabidopsis thaliana*. The same phenomenon might occur in the other species, making protoplasts more easily editable than the intact cells. Choi et al. [36] also showed that trichostatin A (TSA; histone deacetylase HDAC inhibitor) treatment increases Cas9 activity in lettuce and tobacco protoplasts in a concentration-dependent manner. Similarly, Liu et al. [37] observed significant differences in the Cas9 activity between the eu- and heterochromatic regions in rice. These observations suggest that chromatin accessibility may be crucial in developing a reliable tool for the evaluation of gRNA/Cas9 constructs. We believe that by transforming the cell suspension with *Agrobacterium*, using the same method that is used routinely for a stable transformation of cereals, we took a step in that direction. We not only eliminated a dosage effect, present in the PEG-mediated delivery, but also used a recipient tissue that is morphologically closer to the immature embryo-derived calli, which are the tissue of choice for the transformation of cereal species.

Both wheat and triticale are complex hexaploid species posing a major challenge in genome editing experiments. However, we conclude that further studies considering a dose effect and chromatin accessibility will become essential, not only for evaluation but also for overcoming low editing efficiencies in these species. Further corroboration of our statements, as well as methodology improvement, that is, shortening procedure duration, will be the priorities of our future work.

## 4. Materials and Methods

### 4.1. Wheat Cell Suspension Culture

Calli derived from wheat anther cultures *cv*. Svilena were selected as the starting material for cell suspension. Calli were cultured on solid 190-2 [38] medium and subcultured every 3–4 weeks until a friable, fast growing, and non-embryogenic callus was selected. To initiate the suspension, 1–2 g of actively growing callus was transferred to a glass flask containing 30 mL of liquid 190-2 medium and homogenized by pipetting and gentle crushing with a pipette tip. The flasks were then placed in the rotary shaker (120 rpm, 16 h photoperiod, 25 °C). After 2 weeks of shaking, half of the liquid medium volume was replaced with the fresh one. Subsequently, ¾ of the suspension volume was replaced by a fresh medium every week. Cell aggregates were homogenized in each subculture by vigorous pipetting.

### 4.2. gRNA/Cas9 Constructs and Transformation

Two gRNAs were designed to target the first (gABA/1/364) and the second (gABA/2/323) exon of the *ABA 8′-hydroxylase 1* gene. The gRNA/Cas9 constructs were enhanced with *three prime repair exonuclease* 2 (*TREX*2), and the green fluorescent protein (GFP) marker. Additionally, two selection agents (hygromycin—Hyg and phosphinothricin—Phos) were tested for gRNA of ABA/1/364 locus, to verify whether the selection strategy might affect transformation success. In total, three constructs were tested, namely: gABA/1/364 Phos, gABA/1/364 Hyg and gABA/2/323 Phos. Both gRNAs used have been previously tested in triticale protoplasts, where they produced the desired on-target mutations with statistically the same efficiency on all subgenomes [5].

To achieve stable transformation of wheat cell suspensions, *A. tumefaciens* strain AGL1 was used. *Agrobacterium* inoculation and co-cultivation were based on a modified protocol described by Kumlehn et al. [39]. Briefly, an actively growing suspension (3–4 days after subculture) was divided into 6-well plates (3 mL) and supplemented with acetosyringone (0.5 mM) 8–12 h prior to inoculation. At inoculation, liquid medium was removed with pipette and replaced with 1 mL of *Agrobacterium* suspension (OD = 1, in 190-2 medium), also supplemented with 0.5 mM acetosyringone. After 48 h of co-cultivation in darkness, the suspensions were washed with fresh medium supplemented with bactericidal antibiotics (Timentin 150 mg/L, Cefotaxim 100 mg/L) and evaluated under a fluorescent stereo microscope for transient GFP expression. Suspensions with visible GFP expression, indicating efficient T-DNA transfer, were incubated for 3 days on a rotary shaker in the medium without selection agent. Next, suspensions were preliminarily selected in liquid media supplemented with hygromycin (20 mg/L) or phosphinothricin (3 mg/L) for 1 week. Finally, transformed suspensions were homogenized once more, placed on a stack of sterile filter papers to remove liquid medium, and transferred to Petri dishes (90 mm) with solid medium supplemented with Hyg (50 mg/L) or Phos (5 mg/L). The rapidly growing aggregates were transferred to fresh solid selection medium for proliferation. For each construct used, we selected three independent transgenic cell lines and subjected them to further genetic analysis.

### 4.3. Evaluation of On- and Off-Target Editing Efficiency

The TRIzol-based method [40] was used for the simultaneous extraction of genomic DNA and total RNA from selected suspension-derived cell lines. The transgenic character of the cell aggregates was verified by reverse transcriptase-PCR amplification of the *Cas*9 transgene. Finally, genomic DNA was used as a template for amplification with A, B and D subgenome-specific primers designed for on- and off-target sites of interest. A- and B-specific primers were identified in our previous work [5], whereas D-specific primers were newly designed (for: 5′GGCCCATCTTCAAGACGCA3′, rev: 5′AGCGTGCTCTTCCTGTTAATTGAAC3′) Amplicons were Sanger-sequenced by an outside sequencing service provider. Modification frequency, i.e., percentage of modified sequences in a given sample, was identified by the decomposition of the quantitative sequence trace data [41] with TIDE on-line software [http://tide.nki.nl accessed on 1 December 2022]. The 5% cut-off was used as a limit of detection for TIDE analysis. Non-transgenic suspension-derived genomic DNA was used as a control template in all experiments.

## Figures and Tables

**Table 1:** Editing efficiency of on-target modifications in the selected wheat cell lines for three tested gRNA genetic constructs on respective wheat subgenomes (A, B, D). ND—editing not detected, Phos—phosphinothricine, Hyg—hygromycin selection.

Cell Line		Subgenome	
	A	B	D
**gABA/1/364 Phos**			
**g1P1**	64.7%	62.8%	27.8%
**g1P2**	84.2%	75.4%	89.4%
**g1P3**	77.5%	ND	97.7%
**mean ± SD**	**75.5 ± 9.9%**	**69.1 ± 8.9%**	**71.6 ± 38.2%**
**gABA/1/364 Hyg**			
**g1H1**	48.1%	ND	6.9%
**g1H2**	40.1%	ND	7.7%
**g1H3**	11%	ND	ND
**mean ± SD**	**33.1 ± 19.5%**	**0%**	**7.3 ± 0.6%**
**gABA/2/323 Phos**			
**g2P1**	76.1%	ND	63.5%
**g2P2**	96.7%	ND	19.5%
**g2P3**	90.9%	ND	33%
**mean ± SD**	**87.9 ± 10.6%**	**0%**	**38.7 ± 22.5%**

## Data Availability

The datasets generated and/or analyzed during the current study are available from the corresponding author on reasonable request.

## Supplementary Materials

The following supporting information can be downloaded at: https://www.mdpi.com/article/10.3390/ijms24032162/s1.
