# Supplementary material for: Evaluation of CRISPR/Cas9 Constructs in Wheat Cell Suspension Cultures
Source: Int J Mol Sci. 2023 Jan 21;24(3):2162. doi: 10.3390/ijms24032162 (PMC9916915; doi:10.3390/ijms24032162)
Supplement: Supplementary file 1 [file ijms-24-02162-s001.zip › ijms-2134957-supplementary.pdf]

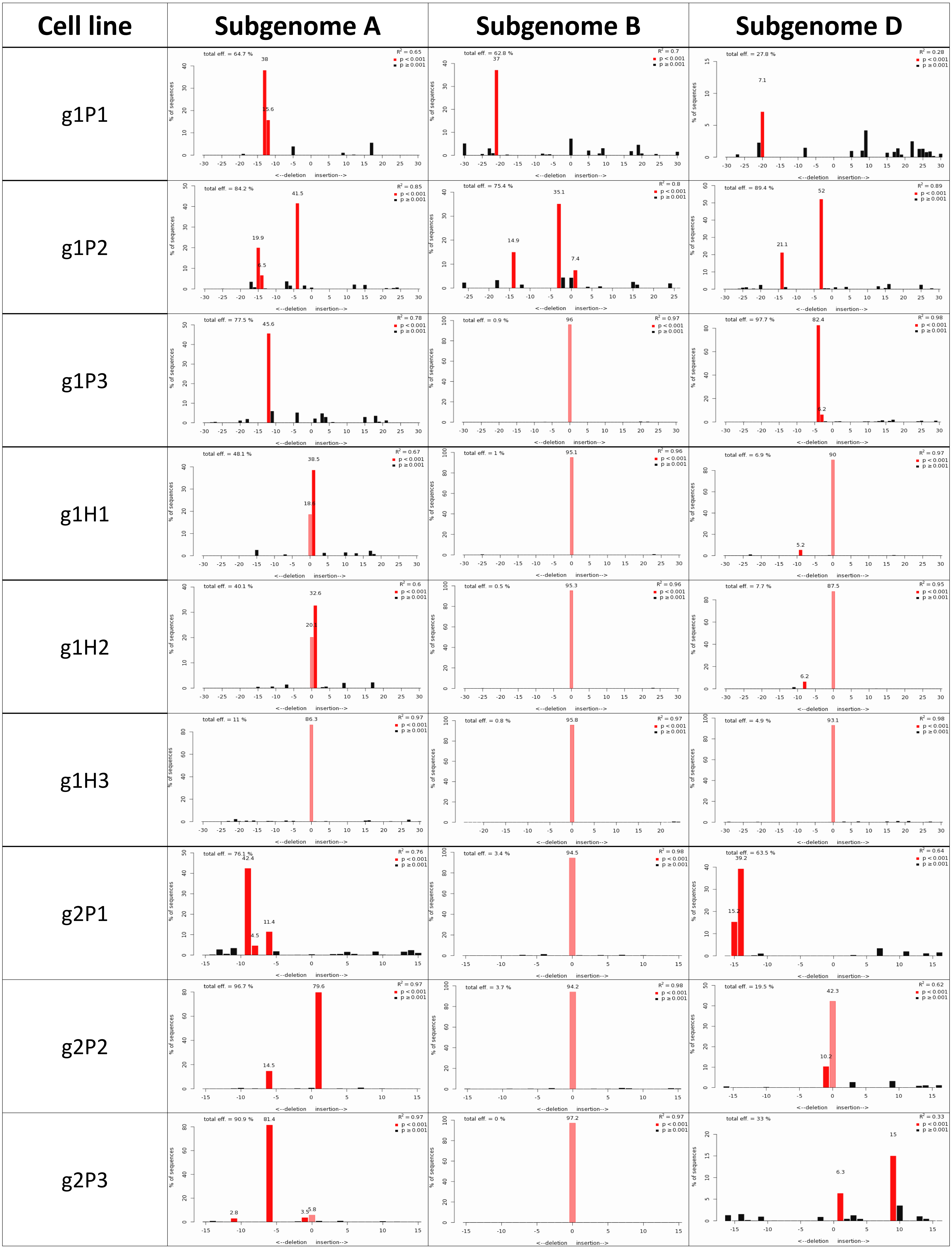

Supplementary Figure S1 – TIDE-generated indel spectra for all tested cell lines. Total efficiency of mutagenesis and frequency of indels shown on particular graphs.
